# Biochar Can Improve Absorption of Nitrogen in Chicken Manure by Black Soldier Fly

**DOI:** 10.3390/life13040938

**Published:** 2023-04-03

**Authors:** Haixu Zhang, Xilu Zhang, Mengxiao Chen, Xin Deng, Yaxin Pei, Jiran Zhang, Hongge Chen, Sen Yang

**Affiliations:** Department of Microbiology, College of Life Sciences, Henan Agricultural University, Key Laboratory of Agricultural Microbial Enzyme Engineering (Ministry of Agriculture), Zhengzhou 450002, China

**Keywords:** chicken manure, black soldier fly, biochar, ammonia

## Abstract

(1) Background: There is growing interest in using insects to treat nutrient-rich organic wastes, such as the black soldier fly (BSF), one of the most efficient organic waste recyclers for upcycling nutrients into the food system. Although biochar (BC) was shown to enhance nutrient retention and the final product quality during the composting of livestock and poultry manure in many previous studies, little information is available on the effect of BC on livestock manure bioconversion by black soldier fly larvae (BSFL). (2) Methods: This study investigated the effect of adding a small amount of BC to chicken manure (CM) on the bioconversion system of the black soldier fly (including N_2_O and NH_3_ emissions and the final distribution of nitrogen during the treatment process). (3) Results: The lowest N_2_O and NH_3_ emission and highest residual nitrogen in the substrate were observed in the 15% BC treatment. The highest bioconversion rate of CM (8.31%) and the peak of larval biomass was obtained in the 5% BC treatment. (4) Conclusions: The results demonstrate the feasibility of adding 5% BC to reduce pollution and achieve a satisfactory BSFL-based CM bioconversion efficiency.

## 1. Introduction

With increasing global demand for protein, the poultry industry is expanding rapidly, leading to the generation of an increasing amount of livestock manure that requires appropriate management. In China, the poultry industry was estimated to have produced 3.16 billion tons of spent litter in 2015 and is increasing at an annual average rate of 2.32%, which has exceeded the rate of industrial solid waste and become the primary pollution source [1,2]. However, poultry litter is a valuable resource, rich in essential plant nutrients, containing 3–5% nitrogen, 0.9–3.5% phosphorus, and 1.5–3% potassium. Moreover, the calcium, magnesium, and sulfur contents are much higher in poultry litter than in cattle or pig manure [2,3]. This indicated that improper disposal of livestock waste will not only lead to the waste of these resources, but also may cause environmental pollution, impacting soil, water, and air quality, possibly exceeding the buffer capacity of the ecosystem, and resulting in the spread of pathogens and other risks [3,4].

Recently, insect bioconversion has been widely recognized as a more sustainable, economically viable, and environmentally friendly solution to livestock and poultry manure management [5,6]. The black soldier fly (BSF) *Hermitia illucens* L. (Diptera: Stratiomyidae) is a promising candidate for treating manure and upcycling it into the food system [7,8]. The black soldier fly larvae (BSFL) can modify the manure’s physical, chemical, and biological properties in one or two weeks and efficiently convert livestock manure into larval biomass and frass, with the former as a feed ingredient in aquaculture and poultry, while the latter as an organic fertilizer [9,10,11]. Additionally, BSFL bioconversion of manure can recycle nutrients, reducing noxious odor, carbon dioxide emission, pathogenic bacteria, and antibiotics [12,13], leading to its rapid use in reducing environmental contamination caused by livestock and poultry manure due to these unique features. Actually, it is being industrialized globally and is recommended as an efficient way to convert organic waste in the new century [12,14]. Despite the advantages of higher efficiency, high product-added value, and lower greenhouse gas (GHG) emissions of BSFL bioconversion versus conventional composting technology, studies have shown that a large amount of nitrogen is still lost in the form of ammonia (NH_3_) and nitrous oxide (N_2_O) during BSFL bioconversion of livestock manure [9,15,16,17]. Additionally, lack of fair exchange of ambient air in the breeding site could lead to NH_3_ enrichment, polluting air quality, possibly negatively affecting larval metabolism, and causing corrosion of rearing equipment [10]. Moreover, N_2_O is considered to be the third most important long-lived greenhouse gas after CO_2_ and CH_4_. [18]. Therefore, reducing NH_3_ and N_2_O emissions is essential for BSFL-based CM bioconversion systems.

Biochar (BC) is a carbon-rich material widely used in environmental applications such as soil remediation, carbon sequestration, and wastewater treatment due to its characteristics, such as large surface area, recalcitrant, and catalysis [19,20]. In recent years, BC has also been widely used as a composting ameliorator to activate other compost materials with horticultural and ecological advantages, including microbial biomass [21,22,23]. In the study of Yin et al. [24], 10% BC treatment was demonstrated to increase the cellulase and urease activities by 56% and 96%. Additionally, BC can change the bacterial community co-occurrence patterns and keystone bacteria and accelerate degradation of carbohydrates and amino acids [25,26,27]. Steiner et al. [28] reported that adding 20% BC to poultry litter compost could achieve faster decomposition, reducing NH_3_ concentration and total nitrogen loss by 64% and 52%, respectively.

As reported by Beesigamukama et al. [29], the BC treatment of brewery-spent grain followed by BSFL rearing could significantly increase larval production and give a 21% increase in nitrogen retention in frass fertilizer, achieving a 195% increase in the biomass conversion rate relative to the control. In the study of Qin, Zhang, Hou, Li, Jiang, Chen, Yu, Tomberlin, Zhang and Li [14], BSFL bioconversion of soybean dregs with BC was shown to reduce NH_3_ emission by 18.0% and increase nitrogen in frass by 35.0%, confirming the feasibility of BC as an amendment for the BSFL-based bioconversion system.

Although BC is commonly used as an amendment in composting to improve aeration condition and reduce nitrogen loss, whether BC can also provide a favorable growth environment and reduce the emission of polluting nitrogen gas during the bioconversion of CM by BSFL is still largely unknown. In order to answer this question, this study investigated the effects of different dosages of BC addition on the bioconversion of CM by BSFL and determined the optimal BC addition amount by comprehensively monitoring BSFL growth, environmental factors, and nitrogen flow directions.

## 2. Materials and Methods

### 2.1. Chicken Manure and Biochar

Chicken manure was obtained from the Henan Agricultural University egg breeding plant (Zhengzhou, Henan, China), with a moisture content of about 75%. Chicken feed formula was 66% corn, 22% soybean meal, 10% wheat bran, 1% rock flour, and 1% premix. Biochar (powder) was purchased from Tianjin Zhiyuan Chemical Reagent Co. (Tianjin, China).

### 2.2. Insect Sourcing

The black soldier fly was the Wuhan strain, which was reared in the microbiology laboratory of Henan Agricultural University. After hatching, the larvae were reared on wheat bran with a moisture content of about 75% for four days under incubation in a plastic box (18 cm × 32 cm × 26 cm) at a temperature of 27.7 ± 1.8 °C.

### 2.3. Experimental Design

To construct the BSFL-based bioconversion of CM with different dosages of BC, dosage of 0% (0 g), 2% (20 g), 5% (20 g), 8% (80 g) and 15% (150 g) (*w*/*w*) BC was stirred into 1000 g of fresh CM in 5 L cylindrical glass bottles (18 cm × 25 cm of diameter × depth) in the treatments of BC0, BC2, BC5, BC8 and BC15, respectively. Three biological replicates were performed for each treatment. After that, each container was inoculated with 500 four-day-old larvae at room temperature for 10 days.

### 2.4. Analytical Methods

The gas detection was performed as reported by Chen, Hou, Pang, Nowar, Tomberlin, Hu, Chen, Xie, Zhang, Yu and Li [16]. To detect NH_3_ emission, the bottle was first sealed. Next, the gas was continuously collected by a gas sampling pump and injected into a 2% boric acid absorbent solution, followed by titration with the standard hydrochloric acid. The gas in the glass bottle was collected in a collection bag, and the N_2_O concentration in the sample was measured within 24 h by gas chromatography reported by Parodi, Gerrits, Van Loon, De Boer, Aarnink, and Van Zanten [9].

The substrate temperature was measured every morning, and the ambient temperature was recorded simultaneously. The substrate in each replicate was mixed, and 15 g (*w*/*w*) of each substrate sample was collected on day 2, 4, 6, 8, and 10 during the experiment. All samples were stored at −20 °C immediately after collection for subsequent analysis.

As previously reported [30], the inorganic nitrogen (NH_4_^+^ and NO_3_^−^) in each sample was measured by the potassium chloride solution extraction-spectrophotometric method (630 nm and 543 nm, respectively). Firstly, each freeze-dried sample (5 g) was mixed into a 2 mol L^−1^ KCl solution (1:10 *w*/*v* ratio) and then detected according to the Chinese national standard HJ634-2012. Another 5 g (*w*/*w*) freeze-dried sample was ground to pass a 1 mm screen, followed by adding concentrated sulfuric acid and H_2_O_2_, and then digestion at 380 °C. After digestion, the total Kjeldahl nitrogen (TKN) content was determined in an automatic TKN tester following the Chinese national standard NY 525–2021.

To determine pH and electrical conductivity (EC), all the samples were thawed and air-dried at room temperature, followed by grinding each sample to pass a 1 mm screen, adding each sample (5.00 g) to 50.0 mL of water with carbon dioxide removed, shaking for 3 min, and standing for 30 min. Finally, each sample’s pH or EC values were measured with a pH acidity and conductivity meter following the Chinese national standard NY 525–2021 [31].

During BSFL bioconversion, 100 larvae were randomly withdrawn, cleaned, and weighed daily to calculate the wet weight of individual larvae. After the BSFL bioconversion, the larvae and residues were dried at 105 °C and weighed to obtain the dry weight. To measure the nitrogen content in the larvae, the harvested larvae (10 g for each treatment) were freeze-dried and then ground into powder [14]. Due to their high-fat content, they were ground three times with the same mill but without a screen, following the Chinese national standard GB/T 6432–2018.

### 2.5. Calculations and Statistical Analysis

The survival performance was calculated by Equation (1):

Survival rate = S_2_/S_1_ × 100% (1)

S_1_ is the total larvae count at the end of the experiment; S_2_ is the initial total larvae count. The reduction rate, bioconversion rate, food conversion rate (FCR), and conversion efficiency of ingested food (ECI) were calculated separately by Equations (2)–(5):

Reduction rate = (m_1_ − m_2_ − m_3_)/m_1_ × 100% (2)

Bioconversion rate = (m_5_ − m_4_)/m_1_ × 100% (3)

FCR = (m_6_ − (m_7_ − m_8_))/(m_9_ − m_10_) × 100% (4)

ECI = (m_5_ − m_4_)/(m_1_ − m_2_ − m_3_) × 100% (5)

where m_1_ is CM dry weight; m_2_, the final residue dry weight; m_3_, BC dry weight; m_4_, initial larvae dry weight; m_5_, final larvae dry weight; m_6_, CM weight; m_7_, final residue weight; m_8_, BC weight; m_9_, initial larvae weight; m_10_, final larvae weight. Because BC is generally considered a resistant material and cannot be biodigested [32], its weight was removed in calculating the final residue.

The experimental data were statistically analyzed with Excell 2021 software and plotted with GraphPad Prism 9.0. One-way analysis of the differences of varied parameters among multiple samples (more than two) was performed using SPSS 26.0 (SPSS Statistical IBM) and followed by the post hoc Tukey’s test.

## 3. Results

### 3.1. Gaseous Nitrogen Emission

**Figure 1:**
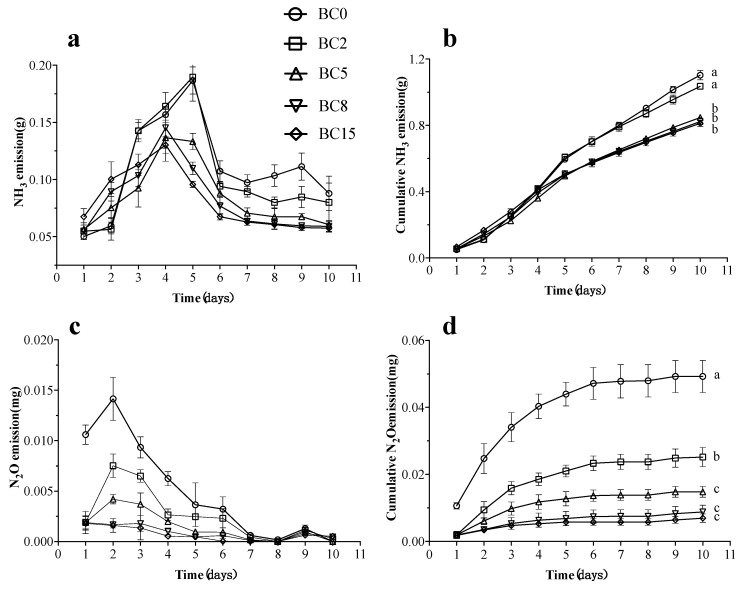
Changes of ammonia (NH_3_) (**a**), cumulative NH_3_ (**b**), nitrous oxide (N_2_O) (**c**), and cumulative N_2_O (**d**) emissions during BSFL bioconversion of chicken manure (CM). Results are presented as the mean ± the standard deviation (SD) of three replicates, with error bars for SD. Different lowercase letters indicated significant differences (*p* < 0.05, post hoc Tukey’s). BC0, BC2, BC5, BC8, and BC15 indicated the treatments of CM amended with 0%, 2%, 5%, 8%, and 15% biochar (BC) (*w*/*w*), respectively.

In this study, BC application was shown to inhibit NH_3_ emission. In Figure 1a, the highest peak of the NH_3_ emission rate was seen to decrease with increasing BC concentration (0.19 ± 0.02, 0.19 ± 0.02, 0.14 ± 0.02, 0.15 ± 0.02, 0.13 ± 0.01 g/day from 0% BC to 15% BC adding dosage, respectively), corresponding to the report of Awasthi et al [33]. Furthermore, the NH_3_ peak appeared earlier in the higher BC treatments (BC5, BC8, and BC15) with a 1 d difference from the BC0 treatment (5 d). Accordingly, with the gradual increase in BC ratio, the final cumulative emission of NH_3_ gradually decreased (1.10 ± 0.05 g, 1.04 ± 0.03 g, 0.85 ± 0.03 g, 0.82 ± 0.03 g, and 0.81 ± 0.03 g, respectively), resulting in a significant difference in BC5, BC8, and BC15 relative to BC0 (*p* < 0.05, Figure 1b).

Additionally, the BC-amended BSFL-based CM bioconversion system was also shown to have a lower N_2_O emission than the BC-free system. At initial bioconversion, the emission of N_2_O showed a similar trend of increasing and decreasing in all the treatments, reaching the highest emission peaks of N_2_O on 2 d (Figure 1c). The final cumulative N_2_O emission values of the BC5, BC8, and BC15 treatments (0.0148 ± 0.0028, 0.0088 ± 0.0035, and 0.0069 ± 0.0022 mg, respectively) are significantly lower than the value of BC2 treatment (0.0252 ± 0.0050 mg) (*p* < 0.05), and very significantly lower than the value of BC0 treatment (0.0493 ± 0.0083 mg) (*p* < 0.01) (Figure 1d). Taken together, the N_2_O emission values are low in all the treatments in the final BSFL-based CM bioconversion system, less than in the system of pig manure [30] and soybean dregs [14], and comparable to the bioconversion system of food waste [9].

### 3.2. Variations of Residual Nitrogen

**Figure 2:**
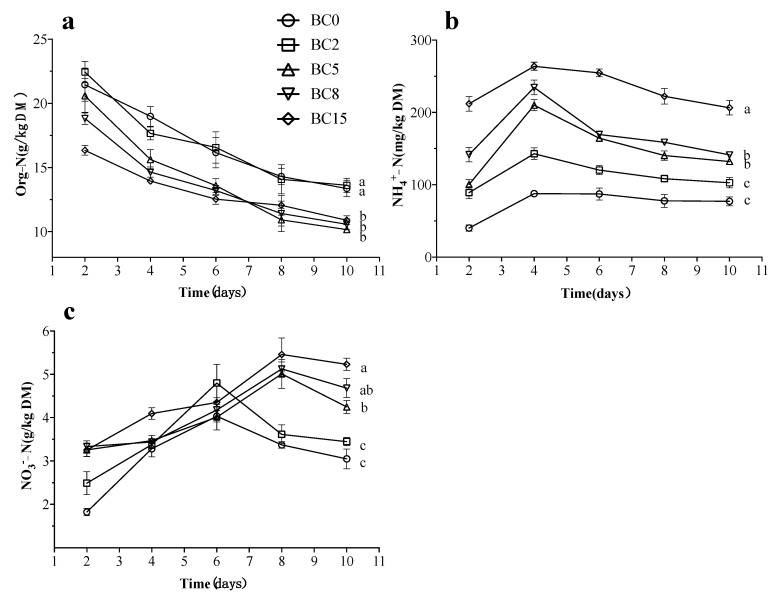
Changes of organic nitrogen (Org-N) (**a**), ammonium nitrogen (NH_4_^+^-N) (**b**), and nitrate nitrogen (NO_3_^−^-N) (**c**) during bioconversion of CM by BSFL. The results are presented as the mean ± the standard deviation (SD) of three replicates, with error bars for SD. DM stands for dry matter basis. Different lowercase letters showed significant differences (*p* < 0.05, post hoc Tukey’s). BC0, BC2, BC5, BC8, and BC15 indicated the treatments of CM amended with 0%, 2%, 5%, 8%, and 15% biochar (BC) (*w*/*w*), respectively.

The variations of the residual nitrogen state were investigated by measuring the contents of organic nitrogen (Org-N), ammonium nitrogen (NH_4_^+^-N), and nitrate nitrogen (NO_3_^−^-N). In the BSFL-based CM bioconversion system, with the bioconversion of the BSFL, the weight of the substrate is decreasing, therefore the dynamic change of the absolute content of nitrogen in the substrate cannot be calculated. However, the Org-N content within the substrate can reflect the degradation rate of the substrate. As shown in Figure 2a, the concentration of Org-N showed a downward trend in all treatments, and finally, in the treatments of BC5, BC8, and BC15 (10.16 ± 0.42, 10.57 ± 0.73 and 10.89 ± 0.60 g/kg, respectively) significantly reduced relative to the treatments of BC0 and BC2 (13.37 ± 1.12 and 13.61 ± 0.96 g/kg, respectively). This showed that adding an appropriate dosage of BC (5% and 8%) could accelerate the decomposition of Org-N in the substrate, while adding a high dosage of BC (15%) seemed to retain more Org-N in the substrate (Figure 2a). 

The NH_4_^+^-N change in the substrate of all the treatments is consistent with NH_3_ emission; with the rapid decomposition of organic nitrogen in the initial bioconversion stage, the NH_4_^+^-N content gradually increased, followed by buffering, all reaching the highest peak on 4 d. The NH_4_^+^-N content in the final substrate was significantly lower in the BC0 and BC2 treatments (77.10 ± 10.68 and 102.84 ± 12.46 mg/kg DM) than in the BC5 and BC8 treatments (132.28 ± 4.44 and 141.27 ± 1.63 mg/kg DM) (*p* < 0.05), and highly significantly lower than in the treatment of BC15 (206.61 ± 17.38 mg/kg DM) (*p* < 0.01) (Figure 2b). This indicated that BC addition to the BSFL-based CM bioconversion system could accelerate the rapid mineralization and ammoniation of organic matter in the substrate.

The proteins in CM were reported to break down into ammonium nitrogen by the BSFL-based bioconversion system in the early stages. Later, nitrification was enhanced in the substrate, converting NH_3_ into NO_3_^−^ [31,34,35]. Because of nitrification, NO_3_^−^-N was continually increased during the early bioconversion stage in all the treatments. At the end of the bioconversion process, the NO_3_^−^-N content was significantly lower in the substrate from the BC0 and BC2 treatments (3.05 ± 0.40 and 3.45 ± 0.16 g/kg DM, respectively) than in the substrate from the BC5 treatment (4.25 ± 0.26 g/kg DM) (*p* < 0.05) and highly significantly lower than in the substrate from the treatments of BC8 and BC15 (4.68 ± 0.38 and 5.23 ± 0.25 g/kg DM, respectively) (*p* < 0.01) (Figure 2c). This indicates that the substrate’s content of NO_3_^−^-N gradually increased with the increase in the BC-adding dosage in the bioconversion system.

### 3.3. Physicochemical Profile Changes of Substrate

**Figure 3:**
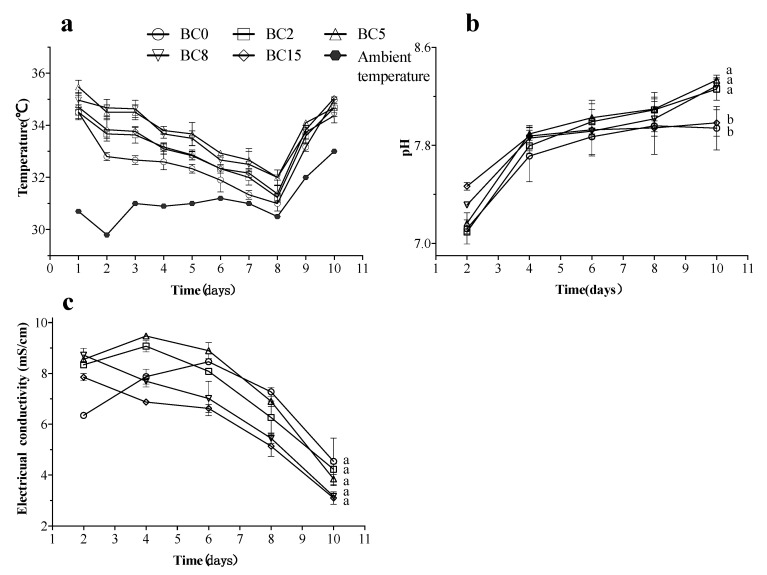
Changes of temperature (**a**), pH (**b**), and electrical conductivity (EC) (**c**) during bioconversion of CM by BSFL. The results are presented as the mean ± the standard deviation (SD) of three replicates, with error bars for SD. Different lowercase letters showed significant differences (*p* < 0.05, post hoc Tukey’s). BC0, BC2, BC5, BC8, and BC15 indicated the treatments of CM amended with 0%, 2%, 5%, 8%, and 15% biochar (BC) (*w*/*w*), respectively.

Temperature is a crucial indicator of the composting process, reflecting the activity of microorganisms and the degradation of organic matter in the substrate [36]. As shown in Figure 3a, in the initial bioconversion stage, the intense oxidation reactions mediated by BSFL and indigenous microorganisms rapidly decomposed the abundant organic biodegradable materials to generate energy, enabling the system at a higher temperature. Moreover, the BC treatment could increase the temperature in the substrate, especially the treatments of BC5 and BC8. Furthermore, the temperature change in the conversion system did not show the typical temperature curve of CM composting, nor did it reach the temperature of around 45–65 °C, the optimum temperature for composting [37], probably due to the small scale of this experiment and lack of ambient temperature control, resulting in a significant environmental influence on the temperature in the bioconversion system.

The pH value of the substrate not only affects the growth of BSFL and gas emission but also indicates the substrate maturity, which is a crucial factor affecting the microbial activities and population community during the composting process [31,38]. Similar pH variation trends were observed in all treatments. Specifically, the decomposition of protein-like organic matter in the system produced a large amount of NH_3_ nitrogen, thus prompting an increase in pH in the initial bioconversion stage. Subsequently, due to the formation and nitrification of low-molecular-weight fats, the pH values increased slowly [31,39]. In Figure 3b, the pH values were seen to vary between 7.0 and 8.5 in all the treatments during bioconversion, consistent with the adaptation range beneficial to the metabolism of the microorganisms [40]. In the present study, the final substrate of the BC2, BC5, and BC8 treatments was generally alkaline (with a pH value of 8.26 ± 0.09, 8.33 ± 0.04 and 8.29 ± 0.06, respectively), which is suitable for the growth of most plants, and significantly higher than the substrate pH of BC0 and BC15 (with a pH value of 7.94 ± 0.18 and 7.98 ± 0.11, respectively) (*p* < 0.05). A possible explanation is that the BC-free treatment (BC0) released a large amount of NH_3_, resulting in a low pH. For the BC15 treatment, due to the high concentration of BC, alkaline substances (NH_4_^+^, etc.) in the substrate could not be desorbed entirely by BC in the detection process. These results indicated that adding 2–8% BC can improve the pH value of the system.

Figure 3c shows the soluble salt contents of the substrate estimated through EC. EC can reflect the salinity of the composting product and its suitability for plant growth and is related to the mineralization of the substrate and the concentration of the mineral fraction [41]. In the early stage, the EC values of the BC0, BC2, and BC5 treatments steadily increased. Subsequently, the EC values began to slow down and decreased after 6 d, probably because BSFL and BC adsorbent absorbed the soluble salts generated by degradation. However, the EC values were continuously reduced in the treatments of BC8 and BC15 from the start of bioconversion. This indicates that adding BC to CM can adsorb soluble salt within the substrate, reduce EC, and accelerate the mineralization of the substrate. At the end of bioconversion, the EC values reached 4.54 ± 1.59, 4.23 ± 0.37, 3.85 ± 0.45, 3.17 ± 0.13, and 3.10 ± 0.43 mS/cm in the treatments of BC0, BC2, BC5, BC8, and BC15, respectively. Despite no significant differences in final EC values among all the treatments, the EC values in the treatments of BC5, BC8, and BC15 met the requirements (EC < 4 mS/cm) for application as tolerable compost products for plants of medium sensitivity [42,43]. 

### 3.4. Bioconversion Performance

**Figure 4:**
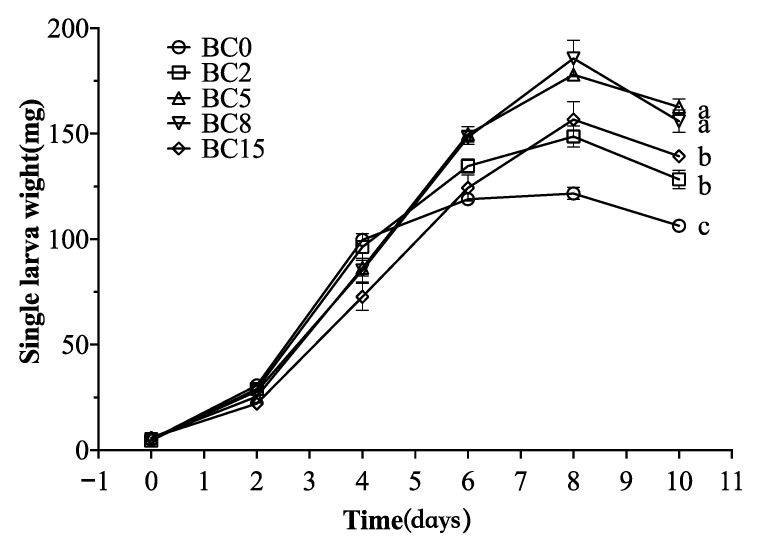
Changes of single larva wet weight during bioconversion of CM by BSFL. The results are presented as the mean ± the standard deviation (SD) of three replicates, with error bars for SD. Different lowercase letters showed significant differences (*p* < 0.05, post hoc Tukey’s). BC0, BC2, BC5, BC8, and BC15 indicated the treatments of CM amended with 0%, 2%, 5%, 8%, and 15% biochar (BC) (*w*/*w*), respectively.

The effects of the BC addition to CM on BSFL were investigated by analyzing the survival rate, single larva wet weight changes, and fresh/dry larval mass. As shown in Table 1, although the larvae survival rate of the BC treatments was higher than that of the BC-free treatment, it did not reach a significant level (*p* > 0.05). This indicated that the BC treatments did not significantly affect the survival rate of larvae. Figure 4 shows the changes in the single larva wet weight with time in all the treatments. During the first 6 d, there were only slight differences in single larva wet weight among all the treatments, followed by the peak on 8 d. The single peak larva wet weight was significantly higher in the treatments of BC5 and BC8 (178.00 ± 3.00 and 185.67 ± 14.98 mg, respectively) than in the treatments of BC2 and BC15 (148.67 ± 8.62 and 156.67 ± 14.74 mg, respectively) (*p* < 0.05) and extremely significantly higher than in the treatment of BC0 (121.67 ± 5.03 mg) (*p* < 0.01). In addition, the final harvested fresh larval mass was significantly higher in the treatment of BC5 (77.15 ± 0.58 g) than in the treatment of BC8 (69.21 ± 4.53 g) (*p* < 0.05) and very significantly higher than in the treatments of BC15 (63.88 ± 2.42 g) (*p* < 0.01) and BC0 (52.80 ± 2.26 g) (*p* < 0.001), ultimately resulting in a significant increase in the dry larval mass of the BC treatments (Table 1). 

The bioconversion performance was investigated by analyzing the reduction rate, bioconversion rate, FCR, and ECI in the different treatments (Table 2). The reduction rate in CM was significantly higher in the treatments of BC5, BC8, and BC15 (59.75 ± 3.63%, 56.93 ± 2.56%, and 58.04 ± 8.47%, respectively) than in BC0 (BC-free treatment) (43.61 ± 0.64%) (*p* < 0.05), with the highest reduction rate for the BC5 treatment. Similarly, the bioconversion rate was significantly higher in the treatment of BC5 (8.31 ± 0.34%) than in the treatments of BC2, BC8, and BC15 (7.51 ± 0.48%, 7.37 ± 0.25%, and 6.87 ± 0.30%, respectively) (*p* < 0.05), and highly significantly higher than in the treatment of BC0 (5.21 ± 0.12%) (*p* < 0.01).

FCR is the most common measure in animal production systems: the amount of feed needed (in kg) to obtain one kg of weight increase in the production animal [44]. In this study, the FCR value was shown to be significantly higher in the treatment of BC0 (12.79 ± 0.21) than in all the BC-addition treatments (9.38 ± 0.81, 8.71 ± 0.38, 9.37 ± 0.69 and 9.41 ± 0.12 from the BC2 to BC15 treatments, respectively) (*p* > 0.05), with no significant difference between all the four BC treatments.

Entomologists commonly use ECI to measure feed conversion efficiency on a dry matter (DM) basis [44], and no significant difference was found in ECI between the five treatments.

### 3.5. Nitrogen Balance

The total TKN in the final substrate and final larvae was measured to evaluate the nitrogen balance, and the nitrogen flow was observed. Table 3 shows the nitrogen distribution, including nitrogen loss, nitrogen in the residue, and nitrogen in larvae. NH_3_ emission was shown as the significant pathway of nitrogen loss, accounting for 16.51–25.90% of total nitrogen in the five treatments. With the increase in the BC-adding dosage, the NH_3_ emission gradually decreased, and BC15 showed the best performance in reducing NH_3_-N loss, from 25.90 ± 0.70% to 16.51 ± 0.92% relative to BC0. Moreover, N_2_O emission accounted for only <0.01% of total nitrogen in all the five treatments, with the lowest N_2_O emission loss for the BC15 treatment. This indicated that BC amendment could effectively decrease NH_3_ and N_2_O emission, and the effectiveness was improved with the increase in the BC-adding dosage, agreeing with the previous study [2]. 

Moreover, a part of the nitrogen in the substrate was bio-converted into the larvae. After bioconversion, the nitrogen accumulated in the larvae was significantly higher in the BC0 treatment (68.88 ± 0.64 g/kg) than in the BC2 treatment (64.48 ± 0.33 g/kg) (*p* < 0.05) and much significantly higher (*p* < 0.01) than in the treatments of BC5, BC8, and BC15 (61.48 ± 1.35, 61.64 ± 0.68 and 60.88 ± 2.14 g/kg, respectively) (Table 3). This indicated that the nitrogen content of BSFL gradually decreased with the increase in the BC-adding dosage. Despite the highest larval nitrogen accumulation in the BC0 treatment, the BC5 treatment had the highest proportion of larval nitrogen to total nitrogen (35.74 ± 0.20%), significantly higher (*p* < 0.05) than that of the BC2 and BC8 treatments (27.53 ± 0.35% and 29.39 ± 1.36%) and much significantly higher (*p* < 0.01) than that of the BC0 and BC15 treatments (23.58 ± 1.28% and 23.59 ± 0.29%) (Table 3). This can be attributed to a significantly higher final harvested dry larval mass in the BC treatments than in the BC-free treatment (Table 1). Additionally, most residual nitrogen was still Org-N, and its proportion in total nitrogen tended to decrease first and then increase with increasing the BC-adding dosage. The Org-N content was the lowest in the BC5 treatment (27.89 ± 0.46%), which was significantly lower (*p* < 0.05) than that in the treatments of BC8 and BC15 (32.23 ± 2.22% and 34.70 ± 0.76%) and much significantly higher (*p* < 0.01) than that in the treatments of BC0 and BC2 (40.95 ± 2.09% and 38.32 ± 1.38%). The proportion of Org-N in residues to total nitrogen showed a significant negative correlation with the proportion of larval nitrogen to total nitrogen, indicating that the larval nitrogen was only derived from the conversion of Org-N in CM (Table 3). Moreover, the proportion of NH_4_^+^-N and NO_3_-N in residues to total nitrogen also gradually increased with the increase in the BC-adding dosage, with the highest proportion in the BC15 treatment (0.96 ± 0.05% and 24.24 ± 0.17%, NH_4_^+^-N and NO_3_^—^N, respectively) and the lowest proportion in the BC0 treatment (0.24 ± 0.03% and 9.33 ± 0.93%, NH_4_^+^-N and NO_3_-N, respectively) (Table 3). These results indicated that BC can strongly retain NH_4_^+^ and NO_3_^−^ from the substrate.

## 4. Discussion

Although the use of BSFL for the bioconversion of livestock manure is considered the most promising treatment method, the nitrogen loss during the conversion process is enormous. In this study, BC was used for the first time as an amendment for the BSFL-based CM bioconversion system, and the BC effect on BSFL performance and nitrogen flow direction during bioconversion was investigated. 

In this study, adding an appropriate proportion of BC to CM was found to be able to accelerate CM degradation, increase the reduction rate and bioconversion rate, reduce the nitrogen loss (NH_3_ and N_2_O), and retain more nitrogen in frass. This can be mainly attributed to the unique properties of BC, such as greater porosity, functional groups, water-holding capacity, cation exchange capacity, and micropore properties [45,46,47]. 

Firstly, the BC effect on the CM bioconversion system is its adsorption function, which is related to the large specific surface area and internal pore volume of BC as well as the presence of various surface acidic functional groups, cation exchange sites, and micropores [21,48,49], enabling the adsorption of NH_3_ released during the bioconversion process and a significant reduction in NH_3_ emission (Figure 1b). Although not considered a GHG, NH_3_ is critical for the agricultural value of the final product and ecosystem protection [50], and its emission is also the primary means for nitrogen loss during aerobic composting or BSFL-based bioconversion [15,51]. Moreover, the content of NH_4_^+^ in the substrate can be a direct indicator of rapid mineralization and ammoniation of organic matter during the compost bio-oxidation stage, reflecting the utilization of nitrogen-containing organic matter [31,32]. In the early stage of bioconversion, the easily degradable organic matter content was relatively high, leading to the rapid reproduction of microorganisms, including ammoniated bacteria, accelerating the decomposition of Org-N and the accumulation of NH_4_^+^ in the substance (Figure 2a,b) [33,34]. However, the production of acidic groups on the surface during BC oxidation, especially carboxyl groups, can effectively adsorb NH_4_^+^ (or its precursor’s urea and uric acid) produced in the system to the BC surface, affecting the NH_4_^+^ and NH_3_ equilibrium and reducing NH_3_ emission, allowing more NH_4_^+^ to be retained in the substrate (Figure 2b) [2,52,53]. 

Secondly, the BC surface is commonly perceived to have a negative charge and cannot retain NO_3_^−^. However, the BC-treated substrates showed higher NO_3_^−^ content (Figure 2c), consistent with the results of Kammann et al. [54], and the NO_3_^−^ retention by BC is probably attributed to non-conventional ion-water bonding/non-conventional hydrogen bonding [55]. Additionally, BC could retain more NH_4_^+^ and NO_3_^−^ in the substrate, thus reducing the pool of inorganic nitrogen available for nitrification and denitrification, leading to a reduction in N_2_O emission (Figure 1d) [56,57]. More importantly, the generated N_2_O can also be adsorbed on the surface by BC and then reduced to N_2_ by oxidation [21,48,58]. Moreover, BC could adsorb Org-N compounds from the substrate through π-bonds, thus reducing the ability to mineralize Org-N compounds, which may be an indirect mechanism for the decrease in NH_3_ emission [21,59,60], and also explains the trend of decreasing and then increasing for Org-N content (Figure 2a), as well as the trend of increasing and then decreasing for nitrogen in larvae with increasing the BC-adding dosage in this study (Table 3). 

Thirdly, BC can also adsorb toxins, heavy metals, and drug residues from CM. Specifically, the toxins can inhibit microbial growth, and heavy metal ions, although not affecting larval weight, can accumulate in larvae and frass, leading to a decrease in the quality of the final product and a significant reduction in bacterial diversity in the gut of BSFL [61]. BC can also adsorb pesticide residues in the substrate that are harmful to BSFL growth, such as cyromazine, thus improving the survival rate and bioconversion rate of BSFL [62].

Fourthly, BC possess large pores, which could improve the substrate’s physical properties, enhancing aeration and gas diffusion, and avoiding the formation of anaerobic microsites [63]. Oxygen content plays a crucial role in the composting process and is a critical factor in the degradation of organic substrates of mixtures by aerobic microorganisms [64]. BC addition into the mix was observed to enhance the effectiveness of oxygen supply during the composting process due to its higher porosity and larger specific surface area [45,64,65]. The increase in the oxygen content in the substrate could enhance the nitrification process of NH_3_ to NO_3_^−^ since nitrifying bacteria are aerobic microorganisms and their proliferation could significantly reduce the loss of gaseous NH_3_ and increase the NO_3_^−^ content, achieving the conservation of nitrogen, despite the release of N_2_O in this process [49,66,67]. Moreover, the increased oxygen content could also change the structure or the activity of the microbial community in the substrate, increasing microbial diversity and reducing pathogenic bacteria [68,69], accelerating the degradation of organic matter during the process and decomposing complex biopolymers to more simple organic compounds (amino acids, small carbohydrates, and phenolic compounds) [21], thus improving the growth environment and the uptake of substrate nutrients by BSFL [24,70]. Additionally, alterations in the microbial structure within the substrate could result in improved BSFL performance [5,71], and similar results were also observed in substrates inoculated with functional bacteria [72].

Fifthly, BC can also alter the symbiotic pattern of the bacterial community, especially promoting key bacterial taxa with a high capacity to degrade carbon sources and increasing the activities of cellulase, urease, dehydrogenase, and xylanase [24,33]. Additionally, it was found that adding BC to vermicomposting could increase the enzyme activities of protease and phosphatase, which promotes the substrate’s rapid decomposition. This may explain the significantly higher larval unit weight and harvested larval dry weight in the BC treatments, while there was a lower nitrogen accumulation in larvae in the BC treatments than in the BC-free treatment because BC promotes the decomposition of carbon sources in chicken manure (Table 1). This illustrates that BC, as a carbon-rich material, is extremely stable and does not introduce any additional nutrients to the system due to its ability of being difficult to be decomposed by microorganisms and used by BSFL, but its addition can improve the growth environment of BSFL.

Sixthly, BC addition can also enable the acquisition of higher temperatures in the substrate (Figure 3a), which can be attributed to the ability of BC to reduce heat loss and enhance aeration, thus increasing the number and activity of microorganisms, accelerating the bioconversion of the substrate, and causing the generation of more heat [73,74]. The higher temperatures generated within the matrix may also accelerate the abiotic oxidation process on the BC surface, enabling more hydrophilic functional groups (e.g., carbonyl groups, hydroxyl) to be used for microbial decomposition [75].

Finally, BC addition could also raise the pH of the substrate (Figure 3b) due to the leakage of some soluble alkaline components from BC [73,76]. Although BSFL can adapt to a wide range of pH values, higher pH values were shown to help accelerate BSFL growth, as reported by Pang et al. [77]. Moreover, the mobility of ions (e.g., heavy metals) is usually determined by pH, and the solubility of their toxic metals is reduced in matrices with a higher pH, thus reducing their toxicity when used as fertilizers [45]. More importantly, higher pH usually could increase the conversion of NH_4_^+^ to NH_3_ [9,10]

## 5. Conclusions

In this study, we investigated the effect of BC as an ameliorator on the BSFL-based CM bioconversion system. The results showed that BC could reduce nitrogen loss (NH_3_ and N_2_O) and enhance nitrogen retention in residue, promoting BSFL growth and increasing the bioconversion rate, which can be attributed to BC’s adsorption function and porous structure. Additionally, the BC5 treatment showed the best bioconversion performance, retaining more nitrogen (40.87%) by BSFL, achieving the highest biomass (22.93 g) and bioconversion rate (8.31%), indicating BC can be used as an ameliorator in the BSFL-based CM bioconversion system. This study provided useful information on the use of BSFL in CM bioconversion and laid a technical basis for using CM as the substrate for large-scale BSFL bioconversion. Future studies can focus on ① the effects of different types of BC on the BSFL-based CM bioconversion system; ② the changes of relevant enzymes in the bioconversion system of BSFL after adding BC, and their effects on the system; ③ the effects of BC addition on the microbial community structure in the substrate in the BSFL-based CM bioconversion system.

## Figures and Tables

**Table 1:** The survival rate, fresh larval mass, dry larval mass, and nitrogen accumulated in larvae in different BC treatments at the end of bioconversion.

Treatments	Survival Rate (%)	Fresh Larval Mass (g)	Dry Larval Mass (g)	Nitrogen Accumulated in Larvae (g/kg)
BC0	91.33 ± 4.62 a	52.80 ± 2.26 d	14.57 ± 0.31 c	68.88 ± 0.64 a
BC2	93.33 ± 6.11 a	71.28 ± 1.70 ab	18.83 ± 0.67 b	64.48 ± 0.33 b
BC5	96.67 ± 3.06 a	77.15 ± 0.58 a	22.97 ± 0.91 a	61.48 ± 1.35 c
BC8	94.67 ± 3.06 a	69.21 ± 4.53 bc	20.37 ± 0.67 b	61.64 ± 0.68 c
BC15	93.33 ± 1.15 a	63.88 ± 2.42 c	19.10 ± 0.75 b	60.88 ± 2.14 c

The results are presented as mean ± standard error (*n* = 3). Mean values followed by the same small letter in the same column do not vary significantly (*p* < 0.05).

**Table 2:** Reduction rate, bioconversion rate, FCR (fresh matter basis), and ECI (dry matter basis) in different BC treatments.

Treatments	Reduction Rate of CM (%)	Bioconversion Rate (%)	FCR (kg)	ECI (%)
BC0	43.61 ± 0.64 b	5.21 ± 0.12 c	12.79 ± 0.21 a	11.95 ± 0.41 a
BC2	50.10 ± 2.33 ab	7.51 ± 0.48 b	9.38 ± 0.81 b	13.66 ± 0.97 a
BC5	59.75 ± 3.63 a	8.31 ± 0.34 a	8.71 ± 0.38 b	13.96 ± 1.24 a
BC8	56.93 ± 2.56 a	7.37 ± 0.25 b	9.37 ± 0.69 b	12.98 ± 0.99 a
BC15	58.04 ± 8.47 a	6.87 ± 0.30 b	9.41 ± 0.12 b	12.18 ± 1.05 a

The results are presented as mean ± standard error (*n* = 3). Mean values followed by the same small letter in the same column do not vary significantly (*p* < 0.05).

**Table 3:** Nitrogen distribution in terms of nitrogen loss, residual nitrogen, and larval nitrogen.

Treatments	Nitrogen Loss (%)		Nitrogen in Residue (%)			Nitrogen in Larvae (%)
	NH_3_	N_2_O	Org-N	NH_4_^+^-N	NO_3_^−^-N	
BC0	25.90 ± 0.70 a	0.0012 ± 0.0003 a	40.95 ± 2.09 a	0.24 ± 0.03 d	9.33 ± 0.93 d	23.58 ± 1.28 c
BC2	23.50 ± 0.96 ab	0.0006 ± 0.0001 b	38.32 ± 1.38 ab	0.31 ± 0.05 d	10.33 ± 0.96 d	27.53 ± 0.35 b
BC5	21.50 ± 1.46 bc	0.0004 ± 0.0001 bc	27.89 ± 0.46 d	0.45 ± 0.03 c	14.42 ± 1.08 c	35.74 ± 0.20 a
BC8	19.27 ± 0.93 cd	0.0002 ± 0.0001 c	32.23 ± 2.22 c	0.56 ± 0.03 b	18.55 ± 0.95 b	29.39 ± 1.36 b
BC15	16.51 ± 0.92 d	0.0001 ± 0.0000 c	34.70 ± 0.76 bc	0.96 ± 0.05 a	24.24 ± 0.17 a	23.59 ± 0.29 c

The results are presented as mean ± standard error (*n* = 3). Mean values followed by the same small letter in the same column do not vary significantly (*p* < 0.05).

## Data Availability

The data presented in this study are available from the corresponding author.
